# Accelerated differentiation of human induced pluripotent stem cells into regionally specific dorsal and ventral spinal neural progenitor cells for application in spinal cord therapeutics

**DOI:** 10.3389/fnins.2023.1251906

**Published:** 2023-09-15

**Authors:** Anne Huntemer-Silveira, Dane Malone, Anna Frie, Patrick Walsh, Ann M. Parr

**Affiliations:** ^1^Department of Neuroscience, University of Minnesota, Minneapolis, MN, United States; ^2^Department of Neurosurgery, University of Minnesota, Minneapolis, MN, United States; ^3^Anatomic Incorporated, Minneapolis, MN, United States; ^4^Stem Cell Institute, University of Minnesota, Minneapolis, MN, United States

**Keywords:** spinal cord injury, hiPSCs, sensory recovery, differentiation, regional specificity, neural progenitor cells, dorsal spinal neurons

## Abstract

Spinal cord injury can attenuate both motor and sensory function with minimal potential for full recovery. Research utilizing human induced pluripotent stem cell (hiPSC) -derived spinal cell types for *in vivo* remodeling and neuromodulation after spinal cord injury has grown substantially in recent years. However, the majority of protocols for the differentiation of spinal neurons are lengthy, lack the appropriate dorsoventral or rostrocaudal specification, and are not typically replicated in more than one cell line. Furthermore, most researchers currently utilize hiPSC-derived motor neurons for cell transplantation after injury, with very little exploration of spinal sensory neuron transplantation. The lack of studies that utilize sensory populations may be due in part to the relative scarcity of dorsal horn differentiation protocols. Building upon our previously published work that demonstrated the rapid establishment of a primitive ectoderm population from hiPSCs, we describe here the production of a diverse population of both ventral spinal and dorsal horn progenitor cells. Our work creates a novel system allowing dorsal and ventral spinal neurons to be differentiated from the same intermediate ectoderm population, making it possible to construct the dorsal and ventral domains of the spinal cord while decreasing variability. This technology can be used in tandem with biomaterials and pharmacology to improve cell transplantation for spinal cord injury, increasing the potential for neuroregeneration.

## 1. Introduction

Spinal cord injury (SCI) is associated with profound deficits in sensory, motor, bowel/bladder, and autonomic function. There is presently no cure for SCI, and it is increasingly apparent that therapeutic approaches targeting multiple systems are essential for meaningful recovery (Griffin and Bradke, 2020). For example, the circuitry of the dorsal and ventral spinal cord, representing sensory and motor domains, respectively, is heavily interconnected (Zholudeva et al., 2021). Thus, recovery in one domain may be impaired when the other remains damaged. Despite this, the majority of preclinical and clinical research to date focuses largely on the recovery of motor function alone. Given that sensory dysfunction after SCI is both common and debilitating, this gap in research neglects a critical aspect of the patient experience and may be impeding the potential success of current strategies (Masri and Keller, 2012).

Human induced pluripotent stem cells (hiPSCs) are used for both *in vitro* modeling and clinical therapy after injury. Due to their capacity to proliferate and recapitulate myriad cell types, hiPSCs offer a promising strategy for the study of spinal cord dysfunction and the creation of dynamic therapies that can target multiple aspects of the injury (Han et al., 2019; Bai, 2020). Using hiPSC-derived neural progenitor cells, “system-in-a-dish” models allow preclinical study of human tissue in various injury processes (Pollard et al., 2021; Lawson et al., 2023). For clinical applications, stem cell populations have been studied for use in cell replacement therapy, which involves the transplantation of neural progenitor cells to bridge the site of injury and support system wide recovery and regeneration (Fischer et al., 2020). However, while several protocols describe the derivation of ventral spinal interneurons and motor neurons for the study of motor recovery (Trawczynski et al., 2019; White and Sakiyama-Elbert, 2019), only two protocols have been published describing the generation of dorsal spinal neurons, which are necessary to support recovery of sensory circuitry (Gupta et al., 2021; Iyer et al., 2022).

To address the need for clinically relevant and diverse region-specific cell types, we have developed standardized protocols to create hiPSC-derived spinal cord progenitors with high efficiency (Parr et al., 2016; Walsh et al., 2018). This work builds upon established differentiation paradigms that utilize morphogen signaling to direct canonical developmental pathways toward acquisition of a neural fate (Chambers et al., 2009; Dréau and Martí, 2012; Lippmann et al., 2014). Our system advances these currently published methods by providing increased speed and enhanced regional specificity. To achieve this, we utilize a patented neural induction approach that allows for the generation of early neuromesoderm progenitors within 2 days of differentiation and neural progenitor cells in 6 days (US11702629B2; Walsh et al., 2020, 2023). Our previous work has applied this technique to the production of ventral spinal neural progenitor cells (vsNPCs) from hiPSCs and subsequent application in SCI transplantation models (Joung et al., 2018; Patil et al., 2020; Lavoie et al., 2022).

In the present study, we describe the rapid differentiation of regionally specific dorsal spinal neural progenitor cells (dsNPCs) from the same early population as our vsNPCs, allowing us to generate both dorsal and ventral spinal domains from the same parent lineage for downstream applications. We further characterized cell fate transitions for both populations at key time points in multiple cell lines, providing a foundation for replicability lacking in other protocols. This work is the first to apply accelerated differentiation strategies to the generation of multiple spinal cell types from a shared population and will allow for future work incorporating the sensory component of recovery after SCI.

## 2. Materials and methods

### 2.1. Human cell culture and differentiation

Four established hiPSC cell lines from healthy donors were utilized in this study: AISC-0031-035 (Kreitzer et al., 2013; Coriell Institute), TMOi001-A (A18945; Gibco), UMN PCBC16iPS (Walsh et al., 2018), and 30HU-002 (Okita et al., 2011; iX Cells). Cells were maintained on vitronectin (AF-140-09; Peprotech) coated flasks (5 μg/mL) with StemFlex medium (A3349401; Thermo Fisher Scientific) at 37°C in 5% CO_2_ humidified incubators. Media was changed every 1 to 2 days and passaging was performed as previously described and according to manufacturer guidelines (Parr et al., 2016). Briefly, cells were incubated 3–5 min with hypertonic citrate solution, detached, and reseeded as clumps at a ratio of 1:5 to 1:15. For neuronal differentiation, cells were clump passaged to maintain a 1:8 to 1:12 split ratio in 24-well plates. Twenty-four hours after passaging, cells were switched to 500 μL/well of Essential 6 medium (A1516401; Thermo Fisher Scientific) and supplemented with daily growth factors according to the stage of differentiation detailed below.

#### 2.1.1. Accelerated differentiation into cervical neuromesoderm (days 1–2)

For day 1 of differentiation, cells were incubated in Essential 6 Medium with 500 nM LDN-193189 (S7507; Selleckchem) and 100 nM BGJ398 (S2183; Selleckchem) to promote neural induction to a primal ectoderm state. For day 2, approximately 24 h later, medium was exchanged for Essential 6 Medium supplemented with 4 μM CHIR99021 (4423; Tocris), 20 ng/mL fibroblast growth factor 2 (FGF2; 100-18B; Peprotech), and 500 nM A8301 (2939; Tocris).

#### 2.1.2. Differentiation of cervical neuromesoderm into dorsal spinal neural progenitor cells (dsNPCs)

On day 3, differentiation diverged for dorsal and ventral spinal lineages. Dorsal sNPCs received Essential 6 medium with 4 μM CHIR99021, 100 nM BGJ398, 100 nM retinoic acid (RA; R2625; Sigma), and 20 ng/mL bone morphogenic protein 4 (BMP4; AF-120-05ET; Peprotech). Day 4 dorsal sNPCs received Essential 6 medium with 20 ng/mL BMP4 and 100 nM RA. From days 5 to 6, dorsal sNPCs received Essential 6 medium with 20 ng/mL BMP4, 100 nM RA, and 20 ng/mL neurotrophin 3 (NT3; 450-03; Peprotech).

#### 2.1.3. Differentiation of cervical neuromesoderm into ventral spinal neural progenitor cells (vsNPCs)

On day 3, ventral sNPCs received Essential 6 with 250 nM wntC59 (5148; Tocris Bioscience), 500 nM smoothened agonist (SAG; 11914; Cayman Chemical), and 100 nM BGJ398. Day 4 ventral sNPCs received Essential 6 medium with 10 μM DAPT (2634; Tocris) and 100 nM RA. From days 5 to 6 ventral sNPCs received Essential 6 medium with 10 μM DAPT, 100 nM RA, and 20 ng/mL NT3.

#### 2.1.4. Differentiation of dsNPCs and vsNPCs into mature spinal neurons

Following the 6-day protocol, dsNPCs and vsNPCs were replated separately onto laminin-521 coated (354221; Corning) 24-well plates (5 μg/mL) and maintained with 1 mL/well of Dulbecco’s modified Eagle’s medium (DMEM) F/12 basal (11039-047; Thermo Fisher Scientific) containing N2 (A13707-01; Thermo Fisher Scientific), B27 (17504-044; Thermo Fisher Scientific), 20 ng/mL NT3, 20 ng/mL brain-derived neurotrophic factor (BDNF; 450-02; Peprotech), and 20 ng/mL glial-derived neurotrophic factor (GDNF; 450-10; Peprotech). Media was replaced every other day. Cells were maintained as such until specific time points for later analysis.

#### 2.1.5. Co-cultures

For co-culturing of dsNPCs and peripheral sensory neurons (7009; Anatomic Incorporated), cells were mixed and plated in standard laminin coated plates at a density of approximately 5 × 10^4^ cells per well. Cells were maintained as described above for 5 weeks prior to calcium imaging. Calcium imaging was performed using the Calbryte 520 AM kit (20650; AAT Bioquest) following manufacturer instructions.

### 2.2. Real-time quantitative polymerase chain reaction (RT-qPCR)

Cells from four cell lines were harvested and RNA extracted using TRIzol Reagent following manufacturer guidelines (15596018; Thermo Fisher Scientific). For cDNA synthesis, the ProtoScript First Strand cDNA Synthesis Kit (E6300S, New England Biolabs) with random primers was used. Amplification was performed using qPCR 2X Green Master Mix (42-121PG; Apex) on a Mastercycler RealPlex2 (Eppendorf) with the following program: 95°C 15 min and 40 cycles of 95°C 15 s, 57°C 15 s, and 72°C 30 s. Gene-specific primers were generated using NCBI primer blast and ordered through Integrated DNA Technologies (IDT). All primer sequences used are listed in Supplementary Table 1. Actin and glyceraldehyde-3-phosphate dehydrogenase (GAPDH) were used as reference genes. An undifferentiated hiPSC control condition, referred to as “Day 0,” was included for all conditions except when specific markers could not be detected, in which case cells at either Day 1 or Day 2 were used. Each sample was run in triplicate and raw cycle threshold (Ct) scores were averaged. Ct scores were excluded if melting curve analysis indicated contamination, primer dimers, or erroneous signals. Fold change in gene expression was calculated using the 2^ddCt^ method.

### 2.3. Immunocytochemistry

Confluent hiPSC-derived cultures seeded on glass coverslips were fixed at multiple timespoints with 10% formalin buffered solution for 10 min at room temperature followed by three washes with 1X phosphate-buffered saline (PBS). Cells were permeabilized with 0.2% TritonX-100 in PBS for 15 min, blocked with SEA blocking buffer (37527, Thermo Fisher Scientific) for 1 h, and incubated in primary antibody solution overnight at 4°C. Coverslips were washed three times with 1X PBS and incubated in species appropriate secondary antibodies conjugated to Alexa Fluor 488, 555, or 647 (Thermo Fisher Scientific) at room temperature for 2 h. For nuclei staining, cells were incubated in 4′,6-diamidine-2-phenylindole dihydrochloride (DAPI; 1:1000; Thermo Fisher Scientific) for 5 min and washed two times in 1X PBS. Each staining was performed on three biological replicates per cell line. Staining was performed on all cell lines except AISC-0031-035, which contained an endogenous mRFP tag that interfered with antibody labeling. Negative controls were obtained by omission of the primary antibody. All primary antibodies used are listed in Supplementary Table 2.

### 2.4. Imaging and quantification

Images of adherent hiPSC-derived cells were acquired using a Leica DMI6000B with a DFC365FX camera running Leica Applications Suite-Advanced Fluorescence, Version 3.1.0. Build 8587. Three to four representative fields per coverslip were collected. Images were processed using FIJI (Schindelin et al., 2012). Images were subjected to background subtraction, local intensity thresholding, binary masks created, and segmentation performed with the MorphoLibJ plugin (Legland et al., 2016). To determine the change in fluorescence expression over time, the mean gray value for each region of interest (ROI) was measured and compared. For quantification of expression between dorsal and ventral sNPCs, the positively stained area for each antibody was measured and divided by total DAPI stained area. To determine the percentage of neurons, astrocytes, and oligodendrocytes, manual cell counting was performed.

### 2.5. Statistics

All statistical analysis was performed in GraphPad Prism, Version 9.5.1. All data are presented as mean ± standard error of the mean (SEM). Statistical significance was determined using Student’s *t*-tests for two groups and one-way analysis of variance (ANOVA) for three or more groups with Dunnett’s multiple comparisons *post-hoc* test. Shapiro–Wilk test was applied for testing the normality of the data. Differences with *P* < 0.05 were considered statistically significant.

## 3. Results

### 3.1. Generation of dorsal and ventral spinal neural progenitor cells

**FIGURE 1:**
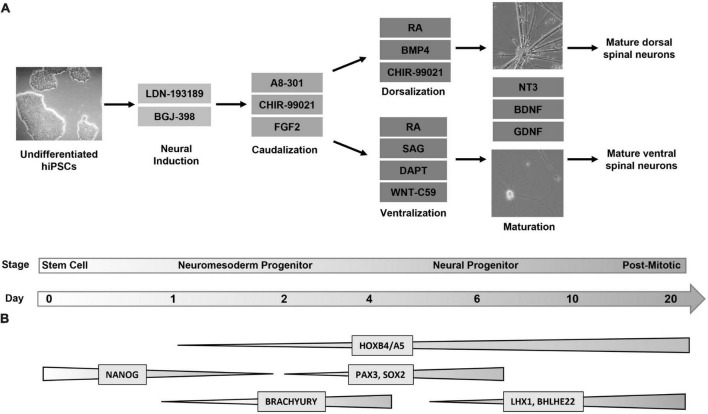
Directed differentiation of dorsal and ventral spinal neurons from a shared lineage. **(A)** Protocol and timeline for the differentiation of dorsal and ventral spinal neurons. Human induced pluripotent stem cells (Day 0, phase contrast image at 5X magnification) undergo a 6-day directed differentiation protocol in which they are exposed to factors to promote neural induction and caudalization. On day 3 the protocol diverges into either dorsal or ventral lineages. Cells express a progenitor identity by day 6 and are then transitioned to a neurobasal maturation media (phase contrast images at 20X magnification) following which they enter a post-mitotic stage at day 20. **(B)** Global changes in gene expression shared by dorsal and ventral populations throughout differentiation. Markers for pluripotency (NANOG) are undetectable by day 2, when caudal markers (BRACHYURY) show increased expression. Dorsal and ventral neurons express a HOX pattern that indicates a cervical to lumbar identity, with the highest expression at the cervical and thoracic levels. Spinal neural progenitors at day 6 express PAX3 and SOX2. Post-mitotic spinal markers LHX1 and BHLHE22 are expressed by both populations. FGF, fibroblast growth factor; RA, retinoic acid; BMP, bone morphogenic protein; SAG, sonic hedgehog agonist; NT3, neurotrophin 3; BDNF, brain-derived neurotrophic factor; GDNF, glial-derived neurotrophic factor.

Four hiPSC lines were plated, differentiated, and harvested at days 0, 1, 2, 6, 10, and 20 in order to characterize gene expression at key developmental transition periods from progenitor to post-mitotic identity. For brevity, the cell lines used will be referred to as follows: Cell Line 1 (AISC-0031-035), Cell Line 2 (TMOi001-A), Cell Line 3 (UMN PCBC16iPS), and Cell Line 4 (30HU-002). Following the first 2 days of differentiation, where cells undergo neural induction and caudalization, cells in each line were then split into two lineages to create the dorsal and ventral populations (Figure 1A). Dorsoventral specification of our cells, similar to other published protocols that incorporate regional specificity, depends on sonic hedgehog pathway mediated ventral specification and bone morphogenic pathway mediated dorsal specification (Dréau and Martí, 2012; Zholudeva et al., 2018; Gupta et al., 2021). Subsequent characterization at each time point was based on established markers of spinal cord development (Alaynick et al., 2011; Sagner and Briscoe, 2019; Li et al., 2023). This revealed global expression patterns shared by both dorsal and ventral populations across all cell lines throughout the course of differentiation (Figure 1B). Cell type specific markers allowed for further classification of specific neuromesoderm (Day 2), progenitor (Day 6), and post-mitotic (Day 20) stages using RT-qPCR and immunocytochemistry and is described below.

### 3.2. Loss of pluripotency and acquisition of early neural identity

**FIGURE 2:**
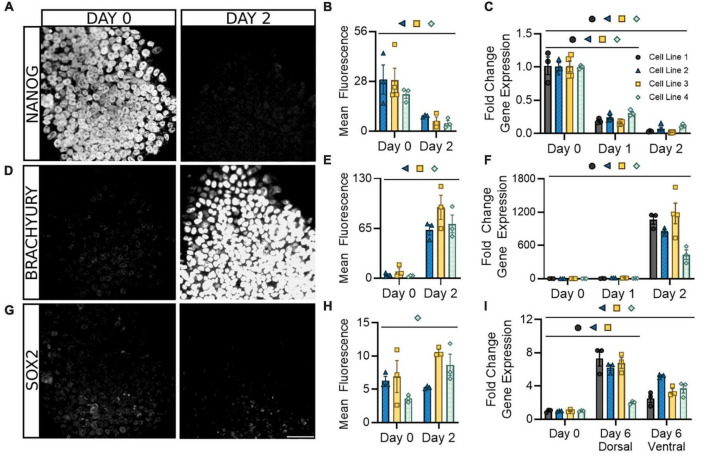
Following neural induction, hiPSCs rapidly exit pluripotency and upregulate genes associated with a spinal neural identity. Expression of the pluripotency marker NANOG is abolished at both the protein **(A,B)** and mRNA **(C)** levels and is barely detectable by day 2. Expression of the caudal marker BRACHYURY increases at the protein **(D,E)** and mRNA **(F)** levels by day 2 of differentiation. Expression of neural progenitor marker SOX2 gradually increases or remains constant at the protein **(G,H)** and mRNA **(I)** level in both dorsal and ventral sNPCs throughout the 6-day differentiation protocol. Representative images from Cell Line 3. Scale bar = 50 um. Quantification of mRNA was performed with RT-qPCR. Dorsal and ventral markers were normalized to undifferentiated hiPSCs at day 0. Significance bar denotes *P* < 0.05 for the corresponding cell line symbol; Student’s *t*-test (Mean Fluorescence) and One-Way ANOVA (Fold Change), *n* = 3–5 biological replicates per cell line. Error bars = SEM. Cell Line 1 = AISC-0031-035, Cell Line 2 = TMOi001-A, Cell Line 3 = UMN PCBC16iPS, and Cell Line 4 = 30HU-002.

We began by assessing the loss of pluripotency and the onset of neurogenic expression in the early stage of differentiation prior to the dorsal/ventral lineage divergence. This protocol utilizes a patented neural induction approach allowing for accelerated transition from the pluripotent state (Walsh et al., 2018, 2023). Within the first 2 days of differentiation, and as early as the first 24 h, there was a significant decrease in both mRNA and protein expression of the pluripotency marker NANOG (Figures 2A–C). This decrease was uniform within and across cell lines. During the same period, there was also a significant increase in mRNA and protein expression of the caudal marker BRACHYURY (*TBXT*), showing similar consistency across cell lines (Figures 2D–F). SOX2 expression, indicative of multipotent neural stem/progenitor cells, gradually increased or remained constant during the first 2 days of differentiation and showed increases up to eightfold by 6 days of differentiation in dorsal and ventral populations (Figures 2G–I). This is consistent with previous literature and supports the acquisition of a neural progenitor identity within 6 days of differentiation (Gupta et al., 2021). Parallel expression of PAX6, which is broadly expressed by spinal progenitors, provides further evidence of this hypothesis (Supplementary Figure 1). Greater variability in SOX2/PAX6 expression observed between cell lines at this stage may indicate subtle timing differences in the point at which this progenitor stage initiates and peaks. Taken together, this demonstrates our differentiation protocol promotes a rapid exit from pluripotency in the first 2 days that corresponds with the adoption of a spinal identity and gradual expression of a neurogenic state observable within 6 days.

### 3.3. Onset of distinct dorsal/ventral spinal progenitor domains

In the developing spinal cord, morphogenic signaling produces eleven transcriptionally distinct neural progenitor populations, termed the cardinal classes, that span the dorsal-ventral axis (Sagner and Briscoe, 2019). We have previously observed the onset of the progenitor stage following the first 6 days of directed differentiation in our vsNPCs (Walsh et al., 2018). At this timepoint, ventral cells show induction of caudal markers predominantly associated with the p2 and pMN domains (Patil et al., 2020). Given this timeline, we next sought to determine whether the dsNPCs follow the same pattern and to characterize the differences in transcriptional profiles between these two populations. While key progenitor markers were broadly detectable from days 4 to 12 in both cell types, expression peaked at day 6, indicating the greatest proportion of progenitor cells present at this stage. Thus, day 6 was selected as the representative timepoint to capture progenitor identity.

**FIGURE 3:**
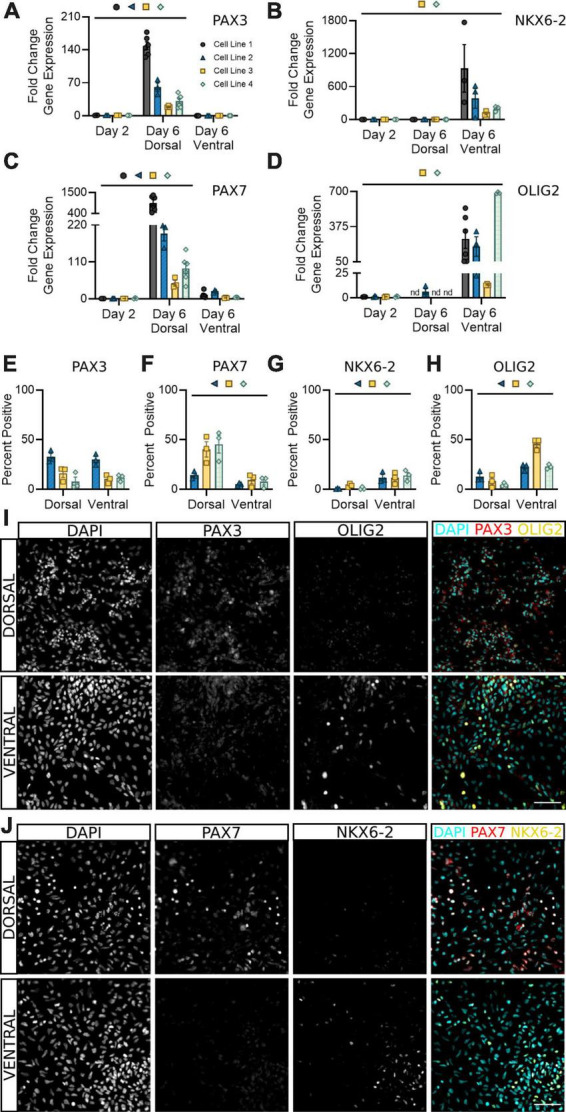
Dorsal and ventral sNPCs begin to express distinct progenitor markers by 6 days of differentiation. Fold change in gene expression of *PAX3* **(A)**, *NKX6-2* **(B)**, *PAX7* **(C)**, and *OLIG2* **(D)** as determined by RT-qPCR in dorsal and ventral sNPCs at Day 6. Markers were normalized to day 2. nd = Not detected. Protein positive area as a percentage of DAPI^+^ fluorescence for PAX3 **(E)**, PAX7 **(F)**, NKX6-2 **(G)**, and OLIG2 **(H)**. Representative images from Cell Line 2 depicting PAX3 and OLIG2 -positive fluorescence in dorsal and ventral cells at Day 6 **(I)** and from Cell Line 3 depicting PAX7 and NKX6-2 -positive fluorescence in dorsal and ventral cells at Day 6 **(J)**. Scale bar = 50 um. Significance bar denotes *P* < 0.05 for the corresponding cell line symbol; Student’s *t*-test (Percent Positive) and One-Way ANOVA (Fold Change), *n* = 3–5 biological replicates per cell line. Error bars = SEM. Cell Line 1 = AISC-0031-035, Cell Line 2 = TMOi001-A, Cell Line 3 = UMN PCBC16iPS, and Cell Line 4 = 30HU-002.

PAX3 is a well-documented marker for spinal neural progenitor cells, with some groups noting it may be more highly expressed in dorsal populations (Alaynick et al., 2011; Gupta et al., 2018; Sagner and Briscoe, 2019; Iyer et al., 2022). Our results confirm these findings at the transcriptional level, with a significant increase in *PAX3* transcripts detectable in dorsal, but not ventral, sNPCs at day 6 (Figure 3A). However, both dorsal and ventral populations at day 6 showed roughly 20–40% PAX3 protein positive expression across all cell lines, supporting attainment of a spinal neural progenitor identity at this time (Figures 3E, I). Differential expression of PAX3, as observed by some groups (Gupta et al., 2018; Sagner and Briscoe, 2019) but not others (Alaynick et al., 2011; Iyer et al., 2022) indicates developmental, timepoint, and likely cell line specific differences in mRNA to protein development that may be important to consider during early characterization of hiPSC-derived sNPCs.

To confirm the onset of regional progenitor identity amongst dorsal and ventral populations, we next quantified expression of the dorsal progenitor marker PAX7, ventral progenitor marker NKX6-2, and premotor neuron (pMN) marker OLIG2. While PAX7 was consistently expressed in dsNPCs (Figures 3C, F, J), NKX6-2 was predominantly expressed in vsNPCs (Figures 3B, G, J). Dorsal progenitor identity was further confirmed by co-expression of MSX1 (Supplementary Figure 2). OLIG2 was enriched in vsNPCs at both the mRNA (Figure 3D) and protein (Figures 3H, I) levels. In contrast, it was detectable only at very low levels or not at all in dsNPCs. Across all cell lines, OLIG2 expression ranges from 25 to 50% in vsNPCs at this timepoint. This may indicate a degree of stochastic variability in the ratio of pMN cells generated by each differentiation that could be targeted to produce higher or lower proportions of motor neuron progenitors. These data support the acquisition of an early spinal progenitor identity with regional specificity in both dorsal and ventral populations within 6 days of differentiation.

### 3.4. Development of post-mitotic interneuron identity

**FIGURE 4:**
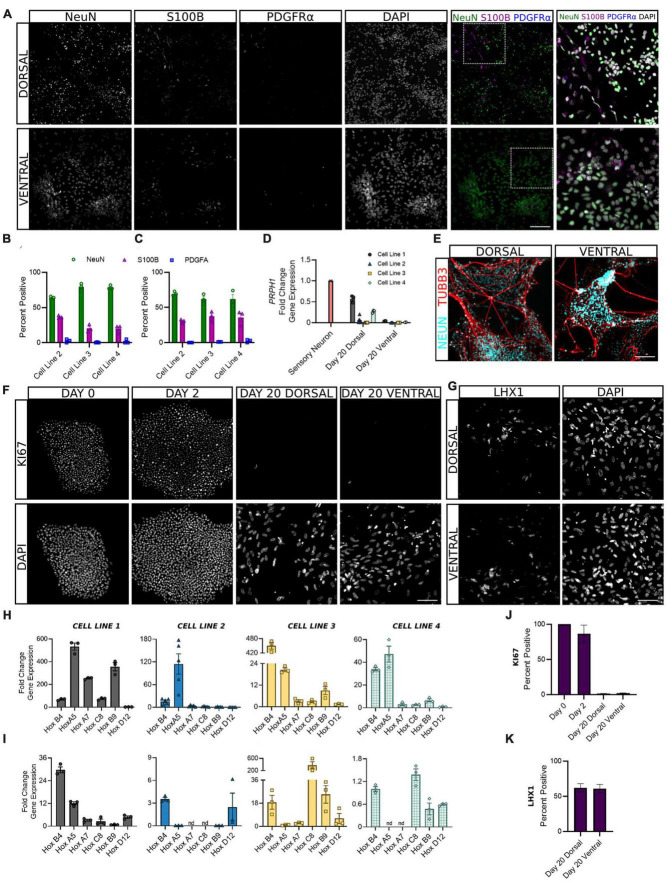
Dorsal and ventral sNPCs develop into mature spinal neurons that express distinct post-mitotic markers following 10 to 20 days of differentiation. Representative images [**(A)**, Cell Line 3] show expression of neural markers NeuN (neurons), S100B (astrocytes), and PDGFRα (oligodendrocytes) in dorsal **(B)** and ventral **(C)** spinal neurons at Day 20. Last column shows inset with DAPI stain. Scale bar = 25 um. **(D)** Dorsal and ventral spinal neurons at Day 20 show decreased fold change in gene expression of the PNS marker *PRPH* compared to peripheral sensory neurons (red). **(E)** NeuN expressing dorsal and ventral spinal neurons co-express TUBB3. Scale bar = 25 um. **(F)** Representative images (Cell Line 3) show KI67 staining at Days 0, 2, and 20 for dorsal and ventral spinal neurons. Scale bar = 50 um. **(G)** Representative images (Cell Line 2) show expression of post-mitotic spinal marker LHX1. Scale bar = 50 um. *HOX* gene profile shows the rostrocaudal identity of dorsal **(H)** and ventral **(I)** cells at Day 20 in all cell lines. Within each graph, HOX genes are ordered rostral to caudal from left to right: *HOXB4*, *HOXA5*, *HOXA7*, *HOXC8*, *HOXB9*, and *HOXD12. ACTIN* was used as a reference gene and *HOX* expression was normalized to Day 2. Percent positive cells for KI67 **(J)** and LHX1 **(K)** combined across cell lines. Error bars = SEM; *n* = 3–5 biological replicates per cell line. Error Bars = SEM; nd = not detected. Cell Line 1 = AISC-0031-035, Cell Line 2 = TMOi001-A, Cell Line 3 = UMN PCBC16iPS, and Cell Line 4 = 30HU-002.

Following the first 6 days of differentiation in which cells were given daily media supplements to direct their development, dorsal and ventral sNPCs were then switched to neurobasal media and maintained through days 10 and 20 to observe the transition to a post-mitotic state. In order to ascertain the expected ratios of mature neural cell types, we quantified the expression of NeuN, S100B, and PDGFRα to evaluate the percentage of neurons, astrocytes, and oligodendrocytes, respectively (Figures 4A–C). At Day 20, 60–80% of both dorsal and ventral cells across all lines expressed NeuN. The majority of remaining cells were positive for the astrocyte marker S100B and 1% or less expressed the oligodendrocyte marker PDGFRα. In addition, to determine the extent to which our protocols may be generating neurons of the peripheral nervous system (PNS), we measured transcriptional levels of the PNS marker peripherin (*PRPH*) at Day 20. Peripherin expression was decreased in both dorsal and ventral neurons across cell lines as compared to levels of expression in peripheral neurons (Figure 4D). While levels of *PRPH* transcript were diminished, expression in dorsal neurons from cell lines 1 and 4 was higher than remaining dorsal and all ventral populations, suggesting a degree of off-target PNS generation in these lines that may be controlled for in the future. TUBB3 was strongly induced by NeuN^+^ dorsal and ventral cells (Figure 4E). Furthermore, at Day 20 fewer than 1% of dorsal and ventral populations were KI67 positive, confirming cells had become post-mitotic (Figures 4F, J).

We also sought to classify the positional identity of our cells along the rostrocaudal axis, which is rarely done in protocols claiming to generate spinal neurons. HOX genes are homeobox genes that specify the rostrocaudal neural axis, predominantly from hindbrain to the lumbosacral spinal cord (Lippmann et al., 2015). An analysis of *HOX* gene expression at Day 20 revealed primarily cervical and upper thoracic identity in both dorsal and ventral populations (Figures 4H, I). *HOXB4* and *HOXA5* showed the most consistent expression across cell lines, suggesting a predominantly rostral spinal identity. However, moderate variability in *HOX* gene expression persisted both within and across cell lines, suggesting that stronger patterning may be incorporated in early stages of the protocol to direct the formation of specific spinal levels.

**FIGURE 5:**
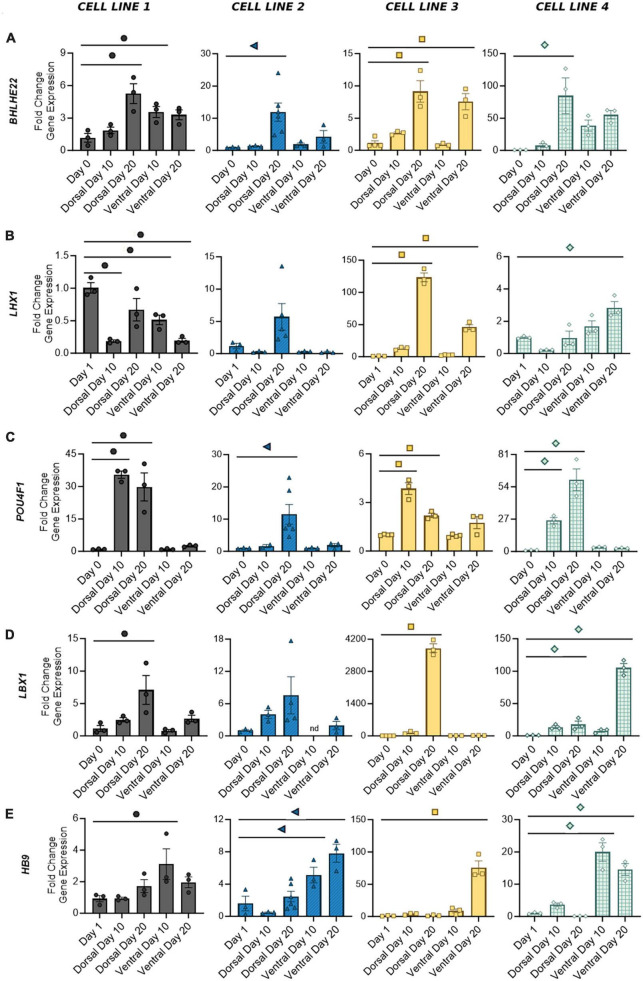
Gene expression of distinct post-mitotic spinal markers in dorsal and ventral cells at Days 10 and 20. Broad spinal markers *BHLHE22* **(A)** and LHX1 **(B)** are expressed by both dorsal and ventral cell types. Dorsal spinal interneuron markers *POU4F1* **(C)** and *LBX1* **(D)** are primarily expressed by mature dorsal populations, whereas motor neuron marker *HB9* **(E)** is expressed by ventral populations. Quantification of gene expression was performed with RT-qPCR and normalized to Day 0 or Day 1 based on the earliest signal detection time point. Significance bar denotes *P* < 0.05 for the corresponding cell line symbol; One-Way ANOVA, *n* = 3–5 biological replicates per cell line. Error Bars = SEM; nd = not detected. Cell Line 1 = AISC-0031-035, Cell Line 2 = TMOi001-A, Cell Line 3 = UMN PCBC16iPS, and Cell Line 4 = 30HU-002.

The adult spinal cord is characterized by six dorsal interneuron (dI) populations and five ventral populations (Sagner and Briscoe, 2019). Thus, in order to identify the distribution of cell types generated by our protocols, we next measured the expression of specific interneuron markers for these populations. Detection of post-mitotic markers began as early as Day 10 and often increased through Day 20 as a greater proportion of cells reached maturity. Expression of genes common to both dorsal and ventral cells was variable across cell lines. *BHLHE22* showed a significant increase in fold change in dorsal and ventral populations in cell lines 1 and 3 and dorsal populations in cell lines 2 and 4 (Figure 5A). *LHX1* showed a significant increase in fold change in dorsal and ventral populations in cell line 3 and ventral populations in cell line 4, and a majority of both populations were LHX1 protein positive as well (Figures 4K, 5B). This supports previous observations that while post-mitotic dorsal and ventral domains are produced, cell line variability contributes to differences in the ratios of specific interneuron populations.

We have previously shown that our ventral populations predominantly express CHX10, indicative of V2 identity, with lower levels of expression associated with other ventral populations (Patil et al., 2020; Lavoie et al., 2022). Similarly, our dorsal populations showed both *POU4F1*, expressed by dI 1-3/5, and *LBX1*, expressed by dI 4–6, suggesting a full complement of dorsal interneurons (Figures 5C, D). As expected, expression of these two markers appears to be mutually exclusive. Cell lines 1 and 3, which showed the most robust fold increase of *LBX1* at Day 20, also showed decreased expression of *POU4F1* from Days 10 to 20. Other groups have demonstrated that BMP driven protocols, such as this one, predominantly drive expression of dI 1–3, while development of dI 4–6 is largely BMP independent (Hernandez-Miranda et al., 2017; Gupta et al., 2018). Thus, as the period of time without BMP treatment increases, some cell lines may be more prone to developmental drift, allowing for acquisition of other dorsal interneuron identities as more cells transition from a progenitor to post-mitotic state. A BMP-free environment may also contribute to the increase in *LBX1* expression seen at Day 20 in the ventral population of cell line 4. This was the only ventral population to show an increase in any of the dorsal markers measured in this study. The motor neuron marker *HB9*, expressed by ventral populations in all cell lines, showed no significant expression in dorsal populations (Figure 5E). Taken together, this demonstrates the development of a mature phenotype in dorsal and ventral cells within 20 days of differentiation, with discrete subpopulations of spinal interneurons detectable as early as 10 days in both protocols.

## 4. Discussion

Regional and functional diversity in the spinal cord arises early in development and produces an intricate mosaic of projection neurons, interneurons, and glial cells that process ascending and descending signals. This complex circuitry poses a major challenge to the application of therapies for spinal cord injuries and diseases. Researchers and clinicians must honor this cellular diversity in order to develop relevant and translational treatments. Growing evidence has demonstrated that patterning along both the rostrocaudal and dorsoventral axis can have a substantial impact on transplant integration, neuroanatomical improvement, and functional recovery (Dell’Anno et al., 2018; Dulin et al., 2018; Zholudeva et al., 2018; Kumamaru et al., 2019). However, cell replacement therapies to date rarely incorporate methods to address the need for level and domain specificity. The lack of appropriate patterning may represent a major barrier that has impeded the success of current cell transplantation approaches for spinal cord injury.

**FIGURE 6:**
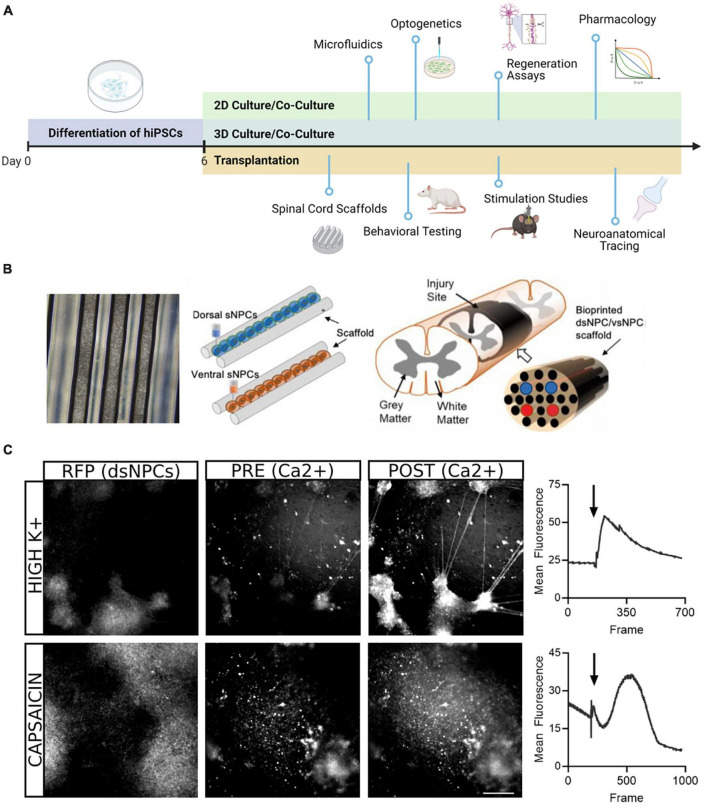
Downstream application of dorsal and ventral spinal neurons. **(A)** Schematic overview of potential uses of dsNPCs and vsNPCs. Following the 6-day differentiation protocol, cells may be replated and matured for long-term 2D or 3D culture in mono- or co-cultures with other relevant cell types. The cultures may be used for numerous *in vitro* assays, including growth assays to evaluate regenerative properties, functional studies using electrophysiological or optogenetic stimulation, or pharmacological experiments to explore synaptic response properties. Dorsal and ventral cells may also be utilized for *in vivo* transplantation into SCI models. Cells may be grafted via injection, scaffold implantation, hydrogel, and many more. Following transplantation, behavioral, electrophysiological, and neuroanatomical analyses may be performed to evaluate the contribution of grafted cells to recovery. Graphic generated using BioRender. **(B)** Schematic of transplantation approach previously utilized by our group in a rodent SCI model. Left: Phase contrast image (5X magnification) of a 5 mm silicone spinal cord scaffold containing 3D printed sNPCs to be used for transplantation. Right: Two scaffolds containing dorsal and ventral populations are sandwiched to approximate the spinal cord and transplanted after transection injury. **(C)** Example of calcium imaging of 5-week co-cultures of dsNPCs expressing RFP and hiPSC-derived peripheral sensory neurons. Top: Following application of high K^+^ media, a detectable increase in calcium fluorescence was detected both populations. Bottom: Following application of capsaicin, a detectable increase in calcium fluorescence was measured in RFP expressing dsNPCs. Scale bar = 200 um.

In the present study, we describe our work generating region specific spinal progenitor cells from hiPSCs that can be utilized for multiple downstream applications (Figure 6). Our rapid induction approach allows us to produce neural progenitors in 6 days and post-mitotic neurons in twenty, while most protocols require a minimum of 30 days (Telias, 2022). For transplantation paradigms where speed and timing are crucial, this improvement has marked relevance for the translatability of these approaches. Our protocol follows conventional methods of differentiation, building upon the seminal work using dual SMAD inhibition for neural induction and subsequent studies demonstrating the roles of BMPs, Wnts, and Shh pathway activators in ensuring the appropriate rostrocaudal and dorsoventral specification for spinal neurons (Chambers et al., 2009; Dréau and Martí, 2012; Lippmann et al., 2015). Adaptation of these factors to our accelerated approach enables the recapitulation of both dorsal and ventral populations from a shared hiPSC lineage in less than 1 month.

The inclusion of the dorsal spinal domain represents a neglected area of research, with the ventral and motor domain being overrepresented in the literature. There are currently very few reports of robust protocols for the derivation of dorsal spinal populations from pluripotent stem cells (Gupta et al., 2021; Iyer et al., 2022). These protocols are lengthy, lack cell line replication, and predominantly rely on human embryonic stem cells for characterization, which face greater challenges with translation than hiPSCs. Gupta et al. (2018, 2021) provide the first major account detailing the production of dorsal interneuron populations, and this work has been groundbreaking for the development of subsequent protocols, including our own. It should be noted that their methodology requires neurosphere formation, which may have contributed to the inability to produce quantifiable biological replicates, as 3D cultures are prone to dynamic changes (Duval et al., 2017). The Ashton group’s protocol is notable in that it produces spinal neurons across both dorsoventral and rostrocaudal domains from adherent cultures (Iyer et al., 2022). Again, however, limited quantification and donor replicability make it unclear whether subpopulations from multiple layers can be reliably produced. Similar to both of these protocols, we achieve dorsal neuron specification through the application of BMPs, and the critical role of these proteins in dorsalization of the developing spinal cord suggests they will continue to be an essential component of future work (Andrews et al., 2017).

Another challenge in the production of dorsal spinal neurons has been the lack of knowledge on the developmental pathways contributing to the formation of each subtype. While the ventral subpopulations may be consistently produced via concentration dependent application of sonic hedgehog pathway activators, only superficial dorsal progenitor layers exhibit a similar response to application of BMP (Jessell, 2000; Andrews et al., 2017; Hernandez-Miranda et al., 2017). No single pathway has yet been identified that can produce all six dorsal populations. BMPs are also an essential component in the production of peripheral sensory neurons and may contribute to off target neural crest formation as seen here. Though quantification of peripheral neuron expression has not been measured in other protocols, we report it here as a fundamental starting point for improvement of dorsal spinal neuron production moving forward. In addition, while landmark gene expression associated with each subtype has been described by several groups, inconsistencies in the timing, duration, and specificity of these markers have contributed to conflicting methods of classification. For example, PAX3 has been described both as a ubiquitous spinal marker and a dorsal specific marker, while MSX1 has been separately associated with roof plate alone, dorsal progenitor populations 1–3 and/or 1–6 (Gupta et al., 2018; Delile et al., 2019; Sagner and Briscoe, 2019; Iyer et al., 2022; Li et al., 2023). Further work must be done to elucidate the precise differences that may contribute to these varied expression profiles. The lack of comprehensive human atlases for spinal cord development complicates this further, with the majority of current work coming from rodent tissue. Human atlases, though limited, were utilized alongside rodent atlases to select markers with the best evidence of representation in the present study (Alaynick et al., 2011; Delile et al., 2019; Li et al., 2023). While the dorsal spinal neurons produced here exhibit expression of markers associated with all mature interneuron subtypes, future analysis is necessary to determine the ratio and consistency of these patterns.

An additional barrier that is common to nearly all cell transplantation studies is a lack of cell line/donor replicates for characterization of the cells produced. While such proof-of-concept studies utilizing only one cell line demonstrate feasibility, this is insufficient to ensure replicability or ultimately translatability. The stochastic nature of stem cell culture and differentiation approaches inherently produces variability that may make analysis and interpretation even within one cell line difficult (Gupta et al., 2018). While this presents a deterrent to including biological replicates, we argue that a thorough understanding of the ways variability arises in differentiation outcomes is necessary to develop improved protocols and to anticipate the differences that may be seen in a human transplantation model. The use of strictly defined reagents as described here is an essential step toward good clinical manufacturing practices and has been shown to improve consistency and minimize line to line variation (Lippmann et al., 2014; Parr et al., 2016; Walsh et al., 2018). In this study, the characterization of four donor cell lines provided unique insights into aspects of our protocol that may be modified to enhance consistency. For example, while neural induction and caudalization to cervical neuromesoderm consistently evoked a robust transition from pluripotency to neural spinal identity, we observed increased variability in dorsal and ventral populations in the expression of subtype specific markers at both the progenitor and post-mitotic state. Furthermore, some cell lines appeared more prone to this drift than others, producing a wider array of spinal subtypes based on mRNA transcript and protein analysis. Understanding the susceptibility of cell differentiation protocols to lineage variability will enhance current efforts toward achieving translatable transplantation paradigms.

For improvement of current cell transplantation methods in the field of SCI, regional cell type specificity is another avenue that has already shown success in enhancing host-to-graft integration, though outcomes of recovery have been almost exclusively locomotor in nature (Dell’Anno et al., 2018; Dulin et al., 2018; Zholudeva et al., 2018). In our own prior work transplanting the ventral spinal neurons described here, we have observed notable anatomical and motor improvement, but locomotor deficits persisted and no measure of sensory recovery was found (Patil et al., 2020; Lavoie et al., 2022). This is true of most transplantation studies that incorporate regional specificity to the ventral spinal domain alone (Erceg et al., 2010; Zholudeva et al., 2018). However, differentiation of hPSC-derived dorsal populations has yet to be performed in SCI models. Considering the prevalence of chronic pain and sensory dysfunction after SCI, there is an immediate need for both *in vitro* and *in vivo* models that can study sensory and pain systems (Masri and Keller, 2012; Han et al., 2019). The dsNPCs described here, which can be produced with vsNPCs from the same source population, are well-suited to future incorporation in cell transplantation models. Furthermore, the cell types produced in this protocol are heterogenous in nature, representing several interneuron classes along the dorsoventral axis as well as supporting glial cells. If specific populations are desired for subsequent use, enrichment or purification strategies may be considered to select for specific populations. Whether it is preferable to produce pure populations or promote cellular diversity in these protocols has been debated by many groups and may ultimately depend on the goals for subsequent application (Astashkina et al., 2012). For transplantation, we have found diverse populations support greater host-transplant integration than pure populations alone, though single cell type grafts may enhance targeting of specific outcomes after injury (Zholudeva et al., 2018; Patil et al., 2020; Lavoie et al., 2022).

The importance of rostrocaudal patterning in promoting transplant integration is not yet well understood. Ensuring grafted cells possess a spinal identity, rather than forebrain, has been shown to improve outcomes after injury (Dell’Anno et al., 2018; Olmsted et al., 2020). Only a handful of studies thus far have generated protocols to specify a cervical, thoracic, lumbar, or sacral identity (Lippmann et al., 2015; Iyer et al., 2022). However, preliminary transcriptional studies have uncovered genetic variations within the cardinal classes of spinal neurons along the rostrocaudal axis (Hayashi et al., 2018; Sweeney et al., 2018). This suggests rostrocaudal patterning may in turn be a critical factor to improve dorsoventral specificity. In the present study, dorsal and ventral cells are patterned to a broadly spinal identity. Our *HOX* gene analysis indicates a predilection toward rostral spinal levels, however, future work may refine our protocols to produce specific levels.

The present study provides a critical step toward the production of consistent differentiation protocols for the generation of hiPSC-derived spinal neurons. These spinal populations have numerous potential applications for both *in vitro* modeling and *in vivo* transplantation studies. With the development of this novel differentiation protocol, we propose the future use of both dorsal and ventral cells in tandem to improve understanding of and outcomes after spinal cord injury.

## Data availability statement

The raw data supporting the conclusions of this article will be made available by the authors, without undue reservation.

## Ethics statement

Ethical approval was not required for the studies on humans in accordance with the local legislation and institutional requirements because only commercially available established cell lines were used.

## Author contributions

AH-S, DM, and AP designed experiments. AH-S, DM, and AF performed experiments. PW and AP provided expertise, consultation, and troubleshooting guidance. AH-S analyzed data and drafted the original manuscript. All authors revised the manuscript and approved the submitted version.
