# Supplementary material for: Accelerated differentiation of human induced pluripotent stem cells into regionally specific dorsal and ventral spinal neural progenitor cells for application in spinal cord therapeutics
Source: Front Neurosci. 2023 Sep 15;17:1251906. doi: 10.3389/fnins.2023.1251906 (PMC10540309; doi:10.3389/fnins.2023.1251906)
Supplement: Supplementary file 1 [file Image_1.PDF]

Supplementary Material

1 Supplementary Figures

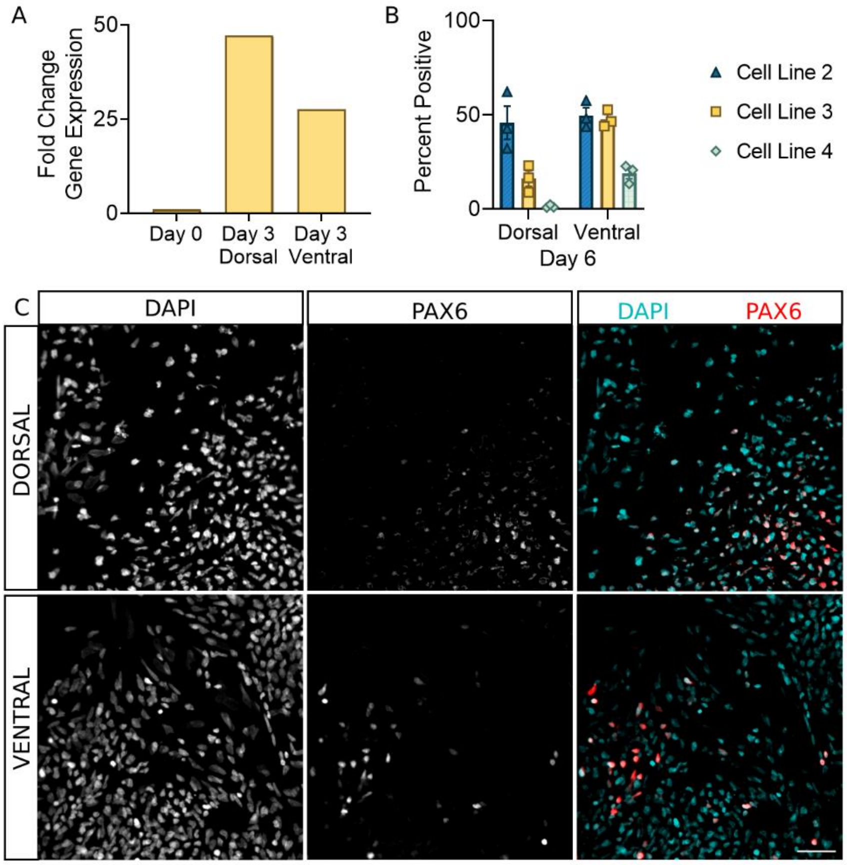

Supplementary Figure 1. Spinal progenitor marker PAX6 is expressed in both dorsal and ventral sNPCs. (A) Fold change in gene expression as determined by RT-qPCR indicates detectable increases in *PAX6* mRNA by day 3 in both dorsal and ventral cells (n=1, Cell Line 3). **(B)** PAX6 positive cells across multiple cell lines (n=3/line) as a percentage of $DAPI^+$ area. Error Bars = SEM. **(C)** Representative images (Cell Line 2) of PAX6 expression at Day 6 in dorsal and ventral populations. Scale bar = 50 um.
