# Supplementary material for: Accelerated differentiation of human induced pluripotent stem cells into regionally specific dorsal and ventral spinal neural progenitor cells for application in spinal cord therapeutics
Source: Front Neurosci. 2023 Sep 15;17:1251906. doi: 10.3389/fnins.2023.1251906 (PMC10540309; doi:10.3389/fnins.2023.1251906)
Supplement: Supplementary file 2 [file Image_2.PDF]

Supplementary Material

1 Supplementary Figures and Tables

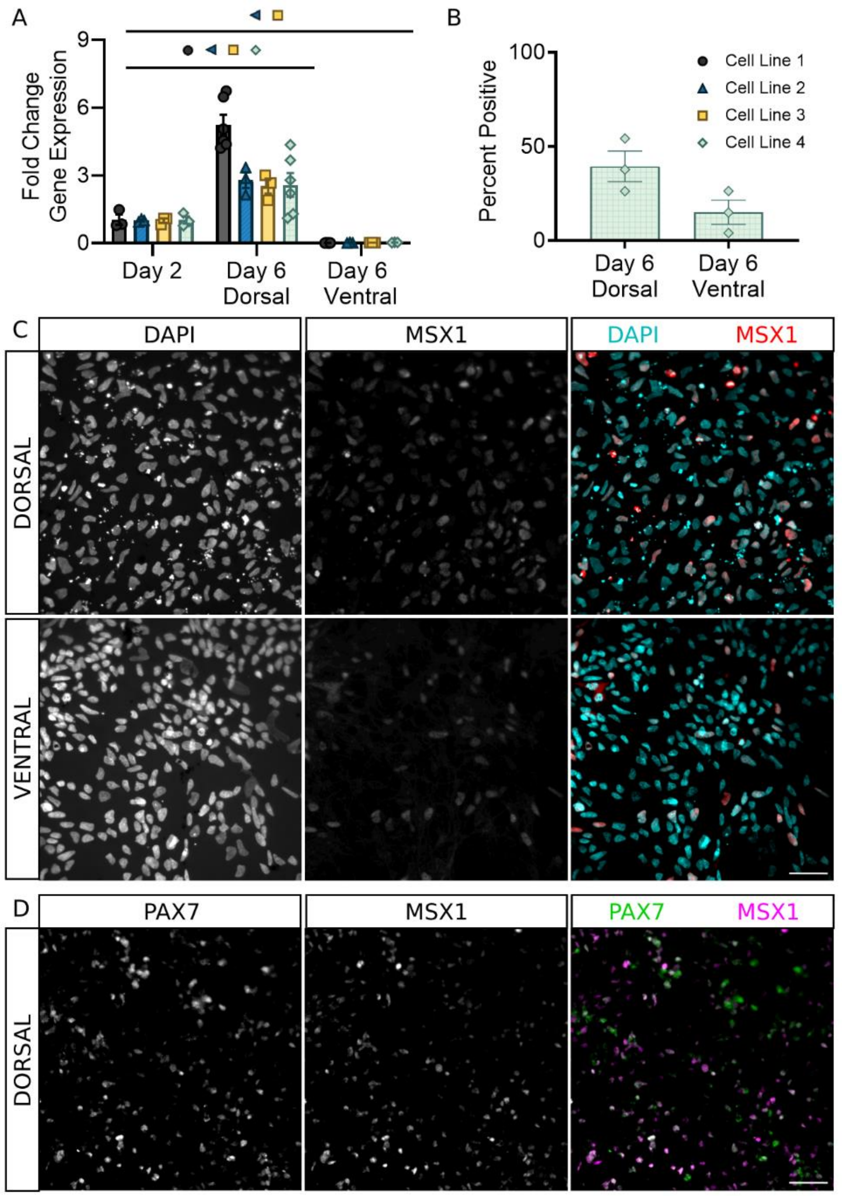

Supplementary Figure 2. Dorsal progenitor marker MSX1 is expressed in dorsal sNPCs. (A) Fold change in gene expression as determined by RT-qPCR indicates detectable increases in MSX1 mRNA by day 6 in both dorsal and ventral cells (n=3-5 per line). While dsNPCs show a significant increase in MSX1 expression, vsNPCs show a significant decrease compared to day two ($p<0.05$, One way ANOVA) **(B)** MSX1 positive cells as a percent of $DAPI^{+}$ area (Cell Line 4). **(C)** Representative images (Cell Line 4) of MSX1 expression at Day 6 in dorsal and ventral populations. **(D)** Representative images (Cell Line 2) showing co-expression of PAX7 and MSX1 in dsNPCs at Day 6. Scale bar = 50 um. Error Bars = SEM.
