# Supplementary material for: Accelerated differentiation of human induced pluripotent stem cells into regionally specific dorsal and ventral spinal neural progenitor cells for application in spinal cord therapeutics
Source: Front Neurosci. 2023 Sep 15;17:1251906. doi: 10.3389/fnins.2023.1251906 (PMC10540309; doi:10.3389/fnins.2023.1251906)
Supplement: Supplementary file 3 [file Table_1.DOCX]

Supplementary Material

# Supplementary Figures and Tables

| **Primer name** | **Primer sequence (5' to 3')** |
| --- | --- |
| *Actin beta-*forward | CCGCCGCCAGCTCAC |
| *Actin beta -*reverse | CTTCTGACCCATGCCCACC |
| *Bhlhb5 (BHLHE22) -*forward | GCCGGCTACCCGTTCAG |
| *Bhlhb5 (BHLHE22) -*reverse | GGGAGCAGCTTTTCGCTTTC |
| *Brachyury (TBXT)* -forward | GGGTACTCCCAATCCTATTCTGACA |
| *Brachyury (TBXT)* -reverse | GGATGGGCAGGCATTCCAAG |
| *Brn3a (POU4F1) -*forward | CAGCAAGCAGCCTCACTTTG |
| *Brn3a (POU4F1) -*reverse | TTGCTCTGCAGCGGCG |
| *Gapdh -*forward | TCCACTGGCGTCTTCACC |
| *Gapdh -*reverse | GGCAGAGATGATGACCCTTTT |
| *HB9 (MNX1) -*forward | TGCCTAAGATGCCCGACTTCA |
| *HB9 (MNX1) -*reverse | GAACCAAATCTTCACCTGGGTCT |
| *HoxA5 -*forward | AGCTGCACATAAGTCATGACAAC |
| *HoxA5 -*reverse | CAATCCTCCTTCTGCGGGTC |
| *HoxA7 -*forward | ACTTCAACCGCTACCTGACG |
| *HoxA7 -*reverse | TCGGACCTTCGTCCTTATGC |
| *HoxB4 -*forward | ACTCCGCGTGCAAAGAGC |
| *HoxB4 -*reverse | AATTGGGTTTTACCGTGCTCA |
| *HoxC8 -*forward | GATGAGACCCCACGCTCC |
| *HoxC8 -*reverse | TTGTCTCTCGGTCAGTCCCA |
| *HoxD12 -*forward | ACTGCCCGACGGCCT |
| *HoxD12 -*reverse | CTTCGTGTAGGGTTTCCGCT |
| *Lbx1 -*forward | CTCGCCAGCAAGACGTTTAAG |
| *Lbx1 -*reverse | TCATACCGTCGCGGCCTTC |
| *Lhx1 -*forward | ACGACTTCTTCCGGTGTTTCG |
| *Lhx1 -*reverse | GGTGAAACACTTTGCTCCGC |
| *Msx1 -*forward | CACTGAGACGCAGGTGAAGA |
| *Msx1 -*reverse | CCAGCTCTGCCTCTTGTAGT |
| *Nanog -*forward | CAATGGTGTGACGCAGGGAT |
| *Nanog -*reverse | GGACTGGATGTTCTGGGTCTG |
| *Nkx6-2 -*forward | CCTTCGAGCAGACCAAGTACC |
| *Nkx6-2 -*reverse | ACCAGACCTTCACCTGGCTCT |
| *Olig2 -*forward | TCGCATCCAGATTTTCGGGTC |
| *Olig2 -*reverse | CCCCAGGGGAAGATAGTCGT |
| *Pax3 -*forward | CAAGCCCAAGCAGGTGACAAC |
| *Pax3 -*reverse | TTTCCCAGCTGAACATGCCC |
| *Pax6 -*forward | CTTCGCTAATGGGCCAGTGA |
| *Pax6 -*reverse | CTCAGATTCCTATGCTGATTGGTGA |
| *Pax7 -*forward | CTTTGGAAGTGTCCACCCCG |
| *Pax7 -*reverse | ACGCGGCTAATCGAACTCAC |
| *Peripherin* -forward | GTGCCCGTCCATTCTTTTGC |
| *Peripherin -*reverse | CTCTGTCACCAGCTCCCCAT |
| *Sox2 -*forward | GGATAAGTACACGCTGCCCG |
| *Sox2 -*reverse | ATGTGCGCGTAACTGTCCAT |

**Supplementary Table 1.** Primer list for RT-qPCR.
