# Supplementary material for: Accelerated differentiation of human induced pluripotent stem cells into regionally specific dorsal and ventral spinal neural progenitor cells for application in spinal cord therapeutics
Source: Front Neurosci. 2023 Sep 15;17:1251906. doi: 10.3389/fnins.2023.1251906 (PMC10540309; doi:10.3389/fnins.2023.1251906)
Supplement: Supplementary file 4 [file Table_2.DOCX]

Supplementary Material

# Supplementary Figures and Tables

| **Antigen** | **Species** | **Dilution** | **Source** |
| --- | --- | --- | --- |
| Β-tubulin III (TUBB3) | Mouse | 1:200 | EMD Millipore (MAB1637) |
| Brachyury | Goat | 1:100 | R&D Systems (AF2085) |
| Ki67 | Rabbit | 1:200 | Abcam (ab15580) |
| Lhx1 | Rabbit | 1:100 | Abcam (ab229474) |
| Msx1 | Goat | 1:200 | R&D Systems (AF5045) |
| Nanog | Rabbit | 1:100 | Proteintech (14295-1-AP) |
| NeuN | Rabbit | 1:200 | Abcam (ab104225) |
| Nkx6.2 | Rabbit | 1:500 | Milipore (ABN1455) |
| Olig2 | Rabbit | 1:200 | EMD Milipore (AB9610) |
| Pax3 | Goat | 1:250 | R&D Systems (AF2457) |
| Pax6 | Mouse | 1:20 | Developmental Studies Hybridoma Bank (AB528427) |
| Pax7 | Mouse | 1:40 | Developmental Studies Hybridoma Bank (AB528428) |
| Pdgfrα | Goat | 1:100 | R&D Systems (AF-307-NA) |
| S100b | Mouse | 1:300 | Millipore (S2532) |
| Sox2 | Mouse | 1:100 | Millipore (MAB4343) |

**Supplementary Table 2.** Primary antibodies for immunocytochemistry.
